# Detection of fungal sequences in human brain: rDNA locus amplification and deep sequencing

**DOI:** 10.1038/s41598-024-82840-7

**Published:** 2024-12-30

**Authors:** Rodrigo Leitao, Iam Ut Wan, Harry Chown, Thomas J. Williams, Matthew C. Fisher, Johanna Rhodes

**Affiliations:** 1 MRC Centre for Global Infectious Disease Analysis, School of Public Health, Imperial College London, London, W12 0BZ UK; 2 Department of Infectious Disease, Imperial College London, London, SW7 2AZ UK; 3 Department of Medical Microbiology, Radboudumc, Nijmegen, The Netherlands

**Keywords:** Microbiology, Molecular biology

## Abstract

The aetiology of Alzheimer’s disease (AD) and Parkinson’s disease (PD) are unknown and tend to manifest at a late stage in life; even though these neurodegenerative diseases are caused by different affected proteins, they are both characterized by neuroinflammation. Links between bacterial and viral infection and AD/PD has been suggested in several studies, however, few have attempted to establish a link between fungal infection and AD/PD. In this study we adopted a nanopore-based sequencing approach to characterise the presence or absence of fungal genera in both human brain tissue and cerebrospinal fluid (CSF). We observed the presence of small fungal burden DNA in two AD brains and a control case (extensive amyloid angiopathy). This approach would be well-placed to investigate potential links between microbial infection and neurodegenerative disease.

**Supplementary Information:**

The online version contains supplementary material available at 10.1038/s41598-024-82840-7.

## Introduction

Alzheimer’s disease (AD) and Parkinson’s Disease (PD) are two of the most common neurodegenerative diseases in the world. Patients with AD or PD display a loss of neurons in the central nervous system (CNS), however, the mechanism by which this occurs has yet to be fully understood^1^. AD is the leading cause of dementia, characterised by progressive cognitive impairment, memory deficits, and loss of independence^2^, with over 520,000 people in the UK being affected by AD-caused dementia^3^. Parkinsonism is a clinical syndrome characterized by slowness of movement, rigidity, tremor, and postural instability^4^. Causes of Parkinsonism may include idiopathic PD and Lewy body dementia (LBD), with PD being the most common form of Parkinsonism^4^ and the second most common type of degenerative dementia in patients aged over 65 years^5^. Currently, identified risk factors include age, diabetes, hypertension, head injury, chronic kidney disease, depression, chronic obstructive pulmonary disease, stroke, genetic susceptibility, and environmental factors. With prevalence of AD, PD, and dementia steadily increasing, investigating further risks underpinning the aetiology of these diseases is crucial as they remain largely unknown^2,6–8^.

Recently, the potential role of microbial infection as an AD/PD risk factor or mediator of progression has gained attention due to the recognition of inflammation as a prominent feature of AD/PD. Additionally, evidence for elevated levels of proinflammatory cytokines in AD and PD patients and increased AD or PD risk conferred by variants in numerous immune-related genes has been shown^9–11^. It has also been suggested that Herpes virus type 1 (HSV1) and *Chlamydia pneumoniae*, and various types of spirochaete are associated with AD^12^. Moreover, a recent study highlighted the association between hospital related infections, such as urinary tract and respiratory infections, with dementia in the elderly population^13^. Mone et al.^14^ have compared bacterial communities in AD and non-AD brains and found differences in bacterial microbiomes, in which the brain microbiome starts off as a healthy microbiome and over time the microbiomes community changes until it becomes associated with AD.

However, in contrast to viruses and bacteria, the role of fungi in AD/PD and dementia has received little attention. The potential involvement of fungi should not be overlooked, as many species are capable of infecting the CNS and neuropathology may be mediated by subsequent inflammation^15^. More direct evidence supporting the potential role of fungal infection in AD/PD originates from a series of studies identifying fungal proteins and DNA in AD/PD patients’ brains and CSF^16–22^. In addition, a study has shown that certain fungal species such as *Saccharomyces cerevisiae*, *Malassezia restricta* and *Candida albicans*, could be associated with certain brain tumors^23^.

Although these findings appear promising, the presence of fungal proteins and DNA in AD/PD brains and CSF is insufficient for establishing the role of infection in the cause or progression of AD/PD as it is unknown whether the fungi were viable and actively contributing to disease pathology^24^. Furthermore, fungal material was also detected in some control individuals^19–21^, which may be expected as increased blood-brain barrier (BBB) permeability is also associated with normal healthy ageing^25^ and there is the possibility of brain microbiomes being present^24^. For these reasons, it is necessary to develop methods to further establish the potential involvement of fungi in the CNS of AD/PD patients and to establish whether the fungi are potentially pathogenic, as well as contributing to neurodegeneration via inflammatory pathways. Our findings suggest that there is a low, but detectable, fungal burden in some human brain samples, regardless of disease status.

## Methods

### Mice tissue

Murine brains were obtained from the Armstrong-James laboratory (Department of Infectious Diseases, Imperial College London). All mouse experiments were approved by the United Kingdom Home Office and the Imperial College London Animal Ethics Committee and performed in accordance with the project license PPL PF9534064. Heterozygote Cftr^tm1Unc^-Tg(FABPCFTR)1Jaw/J mice (originally obtained from Jackson laboratories) were bred in house. All mice were housed in individually vented cages with free access to autoclaved food and water. All methods are reported in accordance with ARRIVE guidelines.

Ex-vivo brain tissue from naive mice was used to optimize the DNA/RNA extraction method, quantitative polymerase chain reaction (qPCR), polymerase chain reaction (PCR) and nanopore sequencing. Briefly, to harvest brain tissue mice were euthanized by cervical dislocation, confirmed through severing the femoral artery. Heads were removed and the scalp was resected. A sagittal incision was carried out from the base of the skull to the tip of the snout. The cranium was then resected, and brains were removed en bloc and cryopreserved at -80 °C until required.

### Human tissue

This project has been approved by Imperial College Healthcare Tissue Bank (ICHTB). Human tissue and CSF were obtained from the ICHTB, the Multiple Sclerosis and Parkinson’s Tissue Bank (PB), and the London Neurodegenerative Diseases Brain Bank (LNDBB) (Table 1). REC3 Wales has confirmed the approval of the ethics for the research project. The data obtained in this work was analysed anonymously. ICHTB is supported by the National Institute for Health Research (NIHR) Biomedical Research Centre based at Imperial College Healthcare NHS Trust and Imperial College London. ICHTB is approved by Wales REC3 to release human material for research (22/WA/0214). PB is approved by Wales REC3 to release human material for research (18/WA/0238). The project is being undertaken solely within the UK and ethical approval for the project is provided under the London Neurodegenerative Diseases Brain Bank Research Tissue Bank ethics approval 18/WA/0206 from Research Ethics Committee for Wales. All research was performed in accordance with the relevant guidelines/regulations and informed consent was obtained from all participants and/or their legal guardians.

**Table 1:** Human brain tissue and CSF obtained from ICHTB, PB and LNDBB.

ID	Sample tissue	Clinical diagnosis	Brain location	Age of donor	Brain supplied
C1	Brain	Normal frozen tissue	NA	59	ICHTB
C2_1	Brain	Normal frozen tissue	NA	30	ICHTB
C2_2	Brain	Normal frozen tissue	NA	30	ICHTB
C2_3	Brain	Normal frozen tissue	NA	30	ICHTB
AD1_1	Brain	Alzheimer’s disease	Cerebellum	86	PB
AD1_2	Brain	Alzheimer’s disease	Frontal cortex	86	PB
C3_1	Brain	Control case (extensive amyloid angiopathy)	Frontal cortex	90	LNDBB
C3_2	CSF	Control case (extensive amyloid angiopathy)	CSF	90	LNDBB
AD2_1	Brain	Alzheimer’s disease	Frontal cortex	87	LNDBB
AD2_2	CSF	Alzheimer’s disease	CSF	87	LNDBB
PD1	Brain	Parkinson’s disease	Frontal cortex		PB
AD3	Brain	Alzheimer’s disease	Frontal cortex	85	PB
C4	Brain	Control case (ovarian cancer)	Frontal cortex	61	PB
PD2	Brain	Parkinson’s disease	Frontal cortex	61	PB
AD4	Brain	Alzheimer’s disease	Frontal cortex	92	LNDBB

*NA* not available.

### DNA extraction

Five different DNA extraction methods were tested in parallel (Supplementary File S1). Amongst these methods, the DNeasy PowerSoil Pro Kit was selected as it provided better performance (Supplementary File S1). DNA was extracted from 25 mg brain samples using the DNeasy PowerSoil Pro Kits (Qiagen, Germany, 47014) following the manufacturer’s protocol with the following modifications: a program with six repeats of bead beating at 4.5 m/s was performed for 45 s using the FastPrep-24™ 5G (Thermo Fisher). Samples were incubated for two minutes on ice after the third repeat. The solution C6 was replaced with elution buffer AE (19077) and the elution volume was decreased to 36 µL with an incubation step of 10 min. Nuclease-free water and PBS buffer were extracted with the samples to control for potential reagent contamination.

### Handling brain and CSF samples in the laboratory

To avoid sample contamination, the handling of human brain and CSF samples, as well as DNA/RNA extractions, were performed in a safety cabinet (Class 2) equipped with a HEPA filter. The safety cabinet, pipettes, tube racks, and other lab consumables were subjected to a treatment of UV light for 30 min before the handling of the samples. In addition, all pipettes and working surfaces were decontaminated with DNA AWAY decontaminant and 10% ChemGene. Disposable sterile blades were used to perform the slicing of the human brains and sterile filtered tips were used to handle the CSF samples. All Eppendorf, PCR tubes, and beads (for bead beating) used in this study were DNA/RNA free to avoid the introduction of contaminants. All DNA and RNA extractions had negative controls (nuclease free-water and PBS) and kit controls.

### Cerebrospinal fluid (CSF)

CSF was extracted by using the DNeasy Blood and Tissue Kit (Qiagen, 69504) with the following modifications: 500 µL of CSF was centrifuged for 10 min at 13,000 rpm and the supernatant (300 µL) was discarded. CSF was then resuspended with remaining fluid (200 µL). The volume of buffer AE was reduced to 36 µL.

### RNA extraction and reverse transcription qPCR (rt-qPCR)

RNA was extracted from 25 mg brain samples using the ZymoBIOMICS™ RNA Miniprep Kit (Zymo Research R2001) following the manufacturer’s protocol with the following modifications: three repeats of bead beating at 4.5 m/s was performed for 45 s using the FastPrep-24™ 5G (Thermo Fisher) and the final elution volume was decreased to 40 µL. Extracted RNA were reverse transcribed into cDNA using the High-Capacity cDNA Reverse Transcription Kit (Thermo Fisher Scientific, 4368814) followed by qPCR (FungiQuant Table 2).

**Table 2:** PCR and qPCR primers used in this study and the respective settings.

Primers sequences (5′–3′)	Master mix reaction	qPCR settings
*Primers 18S rDNA (FungiQuant)*: F-GGRAAACTCACCAGGTCCAG R-GSWCTATCCCCAKCACGA Probe: (6-FAM)-TGGTGCATGGCCGTT-(NFQ-MGB)	10 μL TaqMan™ Universal PCR Master Mix (Applied Biosystems, USA, 4304437) 2 μL forward primer (900 nM) 2 μL reverse primer (900 nM) 2 μL probe (225 nM) 2 μL Nuclease free water 2 μL DNA template/controls	$$\begin{array}{*{20}l} {50\,^{ \circ } {\text{C}}\;{\text{2}}\,{\text{min}}} \hfill \\ {95\,^{ \circ } {\text{C}}\;{\text{10}}\,{\text{min}}} \hfill \\ {\left. \begin{aligned} & {95\,^{ \circ } {\text{C}}\;{\text{15}}\,{\text{s}}} \\ & {60\,^{ \circ } {\text{C}}\;{\text{1}}\,{\text{min}}} \\ \end{aligned} \right\} \times 50}\\ \end{array}$$ Ramp rate: 1.6 °C/s
*Primers ITS region (3.5KB)* (Fig. 1): F-TTTCTGTTGGTGCTGATATTGC GCCAGCAACCGCGGTAA R-ACTTGCCTGTCGCTCTATCTTCT CCTGAGGGAAACTTCG	5 μL sample DNA/controls 45 μL PCR Mix (containing): 10 μL of 5× Phusion HF Buffer (Thermo Scientific, USA, F549L) 1 μL of 10 mM dNTP (Invitrogen, USA, 18427013) 5 μL Forward primer (5 mM) 5 μL Reverse primer (5 mM) 0.5 μL Phusion Hot Start II High-Fidelity DNA Polymerase (2 U/µL) (Thermo Scientific, USA, F549L) 23.5 μL Nuclease free water	$$\left. {\begin{array}{*{20}l} {98\,^{^\circ } {\text{C}}\;30\,{\text{s}}} \hfill \\ {58\,^{^\circ } {\text{C}}\;30\,{\text{s}}} \hfill \\ {72\,^{^\circ } {\text{C}}\;3\,{\text{min}}} \hfill \\ \end{array} } \right\} \times 35$$

### Fungal mock community and spiking mice brains

*Aspergillus fumigatus*, *Candida auris* and *Cryptococcus neoformans* were cultured in Sabouraud Dextrose Agar (SDA) containing chloramphenicol at concentration of 100 mg/L. *A. fumigatus* was incubated for three days at 45 °C, and *C. auris* and *C*. *neoformans* were incubated for five days at 37 °C. After the incubation period, *A. fumigatus*, *C. auris*, and *C. neoformans* were sub-cultured into T25 Nunc EasYFlasks containing SDA. The harvesting of fungal spores for all species was performed by adding 10 ml of sterile 0.1% Tween into the T25 flasks, followed by vigorous agitation and finally filtration through a 10 ml sterile syringe filled with glass wool. Spore counting was performed using a Hemocytometer Counting Chamber (Neubauer-improved bright line Marienfeld, Germany). The spore suspensions of each species were diluted to 2 million spores/ml. A combined 1 million spore (absolute number) mock community containing equal proportions of *A. fumigatus*, *C. auris* and *C. neoformans* was made. To mimic the presence of fungi in infected brains, 25 mg mice brain samples were spiked with 1 ml of 10-fold serial dilutions of the mock community from 10,000 spores to 1 spore (absolute number). Spiked brains were vortexed for 15 s and centrifuged at 13,000 s rpm for 5 min. Supernatant was removed and mice brains were subjected to DNA purification procedures using the DNeasy PowerSoil Pro Kits (Qiagen, Germany, 47014). DNA purification was followed by qPCR and nanopore sequencing.

### Quantitative polymerase chain reaction (qPCR) assays (FungiQuant)

To detect fungal DNA and cDNA in extracted mice and human brain and CSF samples, qPCR was performed using probes and primers (Table 2) targeting a highly conserved region of the fungal 18S ribosomal DNA (rDNA). Sequences were obtained from Liu et al.^26^, and FungiQuant qPCR settings are shown in Table 2.

A standard curve was generated for each 96-well plate using 10- fold serial dilutions of Mycobiome Genomic DNA Mix (ATCC, USA, MSA-1010), containing equal proportions of *A. fumigatus*, *C. neoformans*, *Trichophyton interdigitale*, *Penicillium chrysogenum*, *Fusarium keratoplasticum*, *Candida albicans*, *Candida glabrata*, *Malassezia globosa*, *Saccharomyces cerevisiae*, and *Cutaneotrichosporon dermatis*, ranging from one million to one genome copy equivalence. qPCR on each sample was conducted in triplicate with additional controls with no template (all reaction components excluding DNA) and positive controls (mice brain spiked with *A. fumigatus*, *C. auris* and *C. neoformans*). Data was analysed using the Design and Analysis Software v2.6.0 (Applied Biosystems). In the data analysis, default thresholds were used and the average quantification cycle (Cq) of nuclease-free water, PBS, or the no template control, whichever amplifies earlier, was used as a cut-off to reduce false positive results generated by reagent contamination. Samples were defined as positive for fungi when ≥ 2/3 triplicates demonstrate amplification^26,27^ with Cq values lower than the average Cq of nuclease-free water, PBS, or the no template control.

### PCR barcoding for MinION nanopore sequencing

**Fig. 1:**
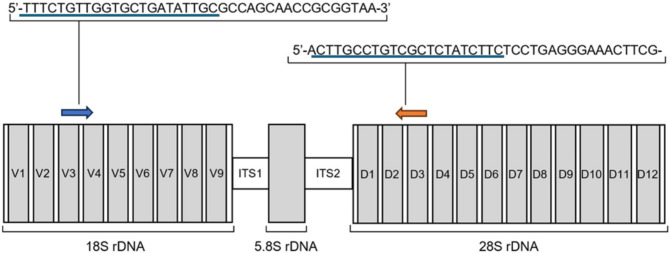
Schematic representation of the fungal ribosomal operon region to be sequenced. Forward (blue arrow) and reverse (orange arrow) primers targeting the fungal ribosomal operon and their sequences are shown. Oxford Nanopore universal tag sequences preceding the 18S and 28S rDNA-specific primer sequences are underlined in blue. The resulting ~ 3.5 kb amplicon covers hypervariable internal transcribed spacer regions 1 and 2 (ITS1 and ITS2), as well as variable domains V3–V9 of the 18S rDNA gene and D1–D3 of the 28S rDNA gene, which can refine taxonomic assignment. Figure modified from D’Andreano and Francino^28^. Presence of PCR products was assessed using 0.5% agarose (Sigma-Aldrich, USA, A9539) gel electrophoresis with SYBR Safe DNA Gel Stain (Invitrogen, USA, S33102), and 1 kb DNA ladders (New England Biolabs, N3232S). Amplicons were subsequently purified using the Genomic DNA Clean and Concentrator®-10 (D4011). Amplicon quantification and size assessment were performed using TapeStation 2200 (Agilent Technologies, G2965A) and the D5000 ScreenTape System (Agilent Technologies, 5067-5588, 5067-5589).

Prior to sequencing, initial PCR was performed on DNA extracted human brain and CSF to amplify fungal targets and attach sequencing barcodes. Primers targeting the fungal ribosomal operon from the V3 region of 18S rDNA to D3 region of 28S rDNA (Fig. 1; Table 2) were used. Sequences were obtained from D’Andreano and Francino^28^ and PCR settings are shown in Table 2. Negative control templates (nuclease free water) were included in all PCR runs.

Barcoding PCR was performed using the PCR Barcoding Expansion 1–12 kit (Oxford Nanopore Technologies, EXP-PBC001). Products from the initial PCR were adjusted to a final volume of 48 µL using nuclease-free water. Only products with sufficiently high concentrations were adjusted to a final concentration of 4.17 nM. Each 100 µL reaction contained 48 µL of PCR product, 2 µL of 10 µM PCR barcode, and 50 µL of PCR mix consisting of 20 µL of 5× Phusion HF Buffer, 2 µL of 10 mM dNTP mix, 1 µL of Phusion Hot Start II High-Fidelity DNA Polymerase, and 27 µL of nuclease-free water. PCR conditions were initial denaturation at 98 °C for 30 s, followed by 15 cycles of 98 °C for 10 s, 62 °C for 15 s, 72 °C for 2 min, and final extension at 72 °C for 10 min. Amplicons were purified using the Genomic DNA Clean & Concentrator®-10 (D4011) and quantified using the broad-range double-stranded DNA assay kit (Invitrogen, USA, Q32853) on the Qubit 2.0 Fluorometer (Invitrogen, USA, Q32866) and TapeStation 2200.

### Library preparation and nanopore MinION sequencing

Barcoded PCR products were pooled in 47 µL of nuclease-free water at equal ratios (totalling 1 µg) and library preparation was carried out using the Ligation Sequencing Kit 1D (Oxford Nanopore Technologies, SQK-LSK109) following manufacturer’s protocols. Samples were quantified by Qubit 2.0 and TapeStation 2200. R9.4.1 flow cells (Oxford Nanopore Technologies, FLO-MIN106.001) were primed and quality controlled following manufacturer’s instructions prior to sample loading. Sequencing was performed using MinKNOW v22.03.5 with 36 h runtime. Fast basecalling and barcoding was enabled and reads with Qscore < 8 were filtered out. Reads longer than 500 bp were retained for downstream analysis.

### Sequence read processing

The quality of raw sequencing reads was assessed using NanoPlot v1.42.0^29^. Sequencing reads then underwent adapter removal using PoreChop v0.2.4^30^ and subsequent quality filtering using NanoFilt v2.8.0^31^. Sequences were retained if they met the following criteria: minimum quality score = 10, minimum length = 500, maximum length = 4000. Reads were then clustered with 90% identity using VSEARCH v2.27.0^32^ set with the “—cluster-fast” and “—strand both” parameters.

### Species assignment

The central sequence was extracted from each cluster using the “split” and “range” functions from SeqKit v2.5.1^33^ to produce representative sequences per cluster. All reads from a cluster were then used to polish the representative sequence to produce a consensus using Medaka v1.11.3 (https://github.com/nanoporetech/medaka). A custom genome database was created by obtaining 9,000 fungal and two human genomes from NCBI^34^ (date accessed: 23/01/2024), covering 5303 fungal species. These were combined and generated into a searchable database using BLAST^35^ v2.15.0. The consensus sequence for each cluster was then searched against the custom database using nucleotide BLAST (90% identity, 90% coverage, 1000 maximum target sequences, one maximum high-scoring pairs). A custom Python v3.11.4^36^ script, incorporating the module ‘Pandas’ v1.5.3^37–39^, was then generated to assign the appropriate taxon to each cluster and obtain the read counts for each respective cluster. The species with the highest bit-score was compared to other alignments that had a bit-score difference less than 40. Using the species names, it was calculated whether alignments were from a single species, genus or from multiple genera and therefore classified as an “Unknown fungal classification”. The taxonomic assignments were collected and visualised in R v4.0.2^40^ using the ‘ggplot2’ v3.4.4^41^, ‘dplyr’ v1.1.4^42^, ‘readr’ v2.1.5^43^, ‘ggsci’ v3.2.0^44^ and ‘forcats’ v1.0.0^45^.

## Results

### Detecting fungal DNA in human samples

No fungal DNA was detected upon performing qPCR assays on all human brain samples and CSF samples. Spiked mice brains with the fungal mock community (*A. fumigatus*, *C. auris* and *C. neoformans*) showed amplification ranging from 100 to 1000 spores (spiked into mice brain). It is worth noting that number of spores spiked into the mice brains is not equivalent to the number of genome copies present in Mycobiome Genomic DNA Mix (ATCC, USA, MSA-1010), shown in the Table 3.

**Table 3:** FungiQuant assay; Cq mean values for mice brain spiking experiment: fungal mock community (*A. fumigatus, C. auris* and *C. neoformans*), and mycobiome genomic DNA Mix (ATCC, USA, MSA-1010).

Samples/standards	Cq mean
1 genome/copy	43.364
10 genome/copies	39.401
100 genome/copies	36.527
1,000 genome/copies	33.946
10,000 genome/copies	30.347
100,000 genome/copies	25.116
1000,000 genome/copies	21.577
10^2^ spiked brain mock community	43.128
10^3^ spiked brain mock community	42.689
10^4^ spiked brain mock community	40.011

Fungal mock community is represented by the total number of spores spiked into the mice brain, whilst the Mycobiome Genomic DNA Mix units are in genome/copies (as stated by the manufacturer).

When spiking the brain with mock community the Cq Mean value for the mice spiked brain with 100 fungal spores is closer to 1 genome/copy than the 100 genome/copies ATCC DNA standard. The limit of detection (LOD) for genomic DNA standards (MSA-1010) used in this assay is one genome copy.

RNA extracted from human brain samples (Table 1) was transcribed into cDNA and a qPCR assay was performed using the 18S primers mentioned above. No fungal cDNA was detected in any of the brain samples (Table 1).

### Species classification

Human brain and CSF samples, mycobiome genomic DNA Mix and “DNA-free” negative controls (BSA and DNA extraction kit control) was sequenced on MinION and analyzed using BLAST to determine which species, if any, are present in AD/PD control brains and CSF. The number of reads for each sample is shown in Table 4.

**Table 4:** Barcode sample ID and total read count, sample type and disease condition (Control = non-diseased brain, Disease = AD/PD brain, negative = negative control, positive = positive control).

Barcode	Total read count	Sample	Sample type	Condition
0	3438	C2.1	Brain	Control
1	3667	C2.2	Brain	Control
10	403	BSA 1	DNA free	Negative
11	23,914	Mycobiome Mix	Mock community	Positive
12	667	PD1	Brain	Disease
13	665	AD3	Brain	Disease
14	461	C4	Brain	Control
15	573	PD2	Brain	Disease
16	749	AD4	Brain	Disease
17	103	BSA 2	DNA free	Negative
18	388	DNA extraction kit control	DNA free	Negative
19	56,262	Spiked DNA	Mock community	Positive
2	1438	C2.3	Brain	Control
3	1393	C1	Brain	Control
4	263	AD1_1	Brain	Disease
5	10,171	AD1_2	Brain	Disease
6	7543	C3_1	Brain	Control
7	285	C3_2	CSF	Control
8	12,307	AD2_1	Brain	Disease
9	2366	AD2_2	CSF	Disease

**Fig. 2:**
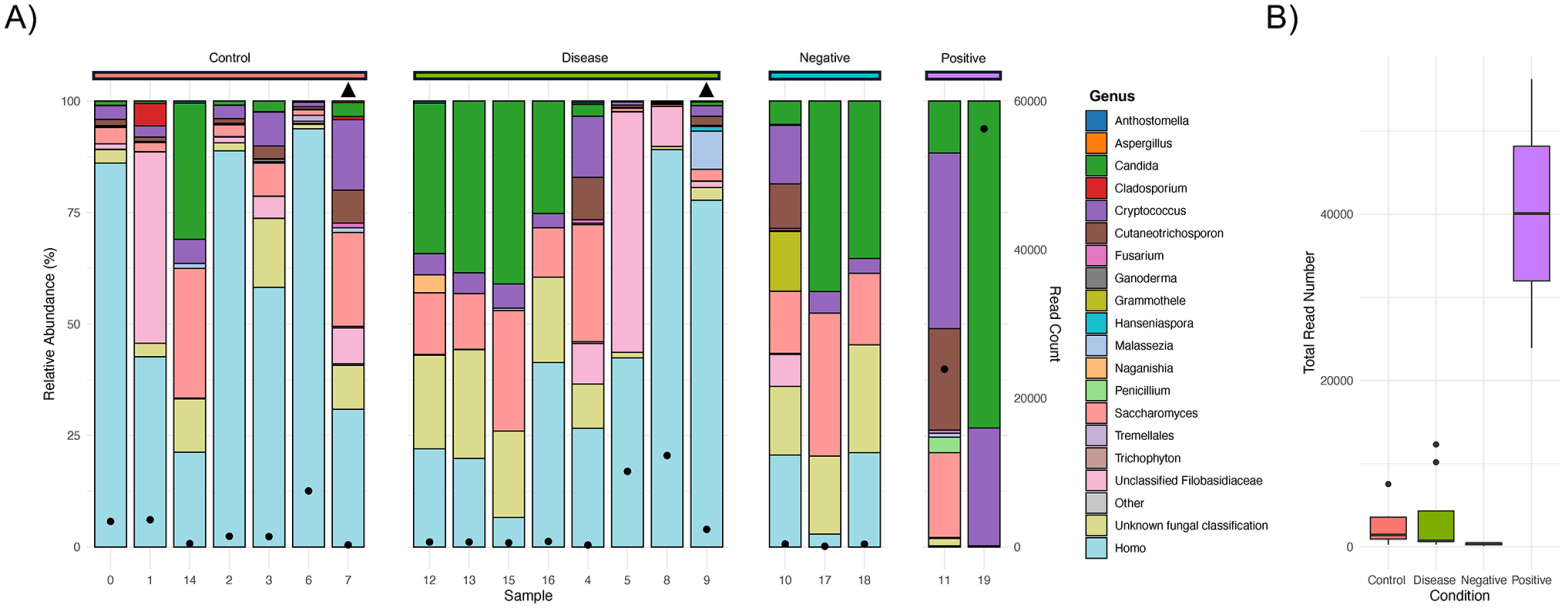
Metabarcoding analysis of samples. (**A**) Genus-level relative abundance of fungi and human (~ 3.5 kb amplicon region) of sequences taken from patients with/without either Alzheimer’s or Parkinson’s (disease and control), alongside analysis of DNA-free standards (negative) and known mock community standards (positive). X-axis indicates each sample, the first y-axis (left-hand side) indicates relative abundance as a percentage and the second y-axis (right-hand side) indicates total read count. Samples marked with a black triangle are from CSF samples, the remaining are from patient brains. Black circles depicts the number of reads per barcode. (**B**) Box-plot depicting total read numbers for each condition.

During the analysis of the metabarcoding data it was observed that DNA-free controls had limited read counts (total read counts ranged from 103 to 403, Fig. 2A; Table 4), whereas known mock communities had a higher read count (ranged from 23914 to 56262, Fig. 2A; Table 4). In addition, disease conditioned brains had higher read counts compared to control brains. However, these counts were a fraction of the positive control (Fig. 2B).

Our nanopore approach was able to correctly identify all ten genera present in the mycobiome genomic standards (barcode 11; Fig. 2A): *Alternaria*, *Aspergillus*, *Candida*, *Cryptococcus*, *Cutaneotrichosporon*, *Fusarium*, *Malassezia*, *Nakaseomyces* (*Nakaseomyces glabratus* previously known as *Candida glabrata*), *Penicillium*, *Saccharomyces* and *Trichophyton* (Supplementary Table S1). In addition, we were also able to detect the three genera, *Aspergillus*, *Candida* and *Cryptococcus*, spiked into the mice brain with a relative abundance of 73.3% for *Candida* and 26.4% for *Cryptococcus* (barcode 19, Fig. 2A) (Supplementary Table S1). However, the number of reads for *Aspergillus* were extremely small, with only three reads for barcode 11 (genomic standards) and four reads for barcode 19 (spiked mice brain with fungal mock community). This could possibly be explained by the high variability within the ITS region; despite this variability making the ITS region an excellent fungal DNA barcode region, it can also create shortcomings due to the large sequence divergences. For instance, ~ 42% of full-length ITS sequences in the International Nucleotide Sequence Database Collaboration (INSDC) do not include the full species names and 29% are only annotated as belonging to the fungal Kingdom (or labelled as uncultured fungus)^46^. In addition, there is a great proportion of fungal sequences in the databases that are incorrectly annotated due to either changes in the taxonomic classification over the years or due to simple human error^47^.

**Fig. 3:**
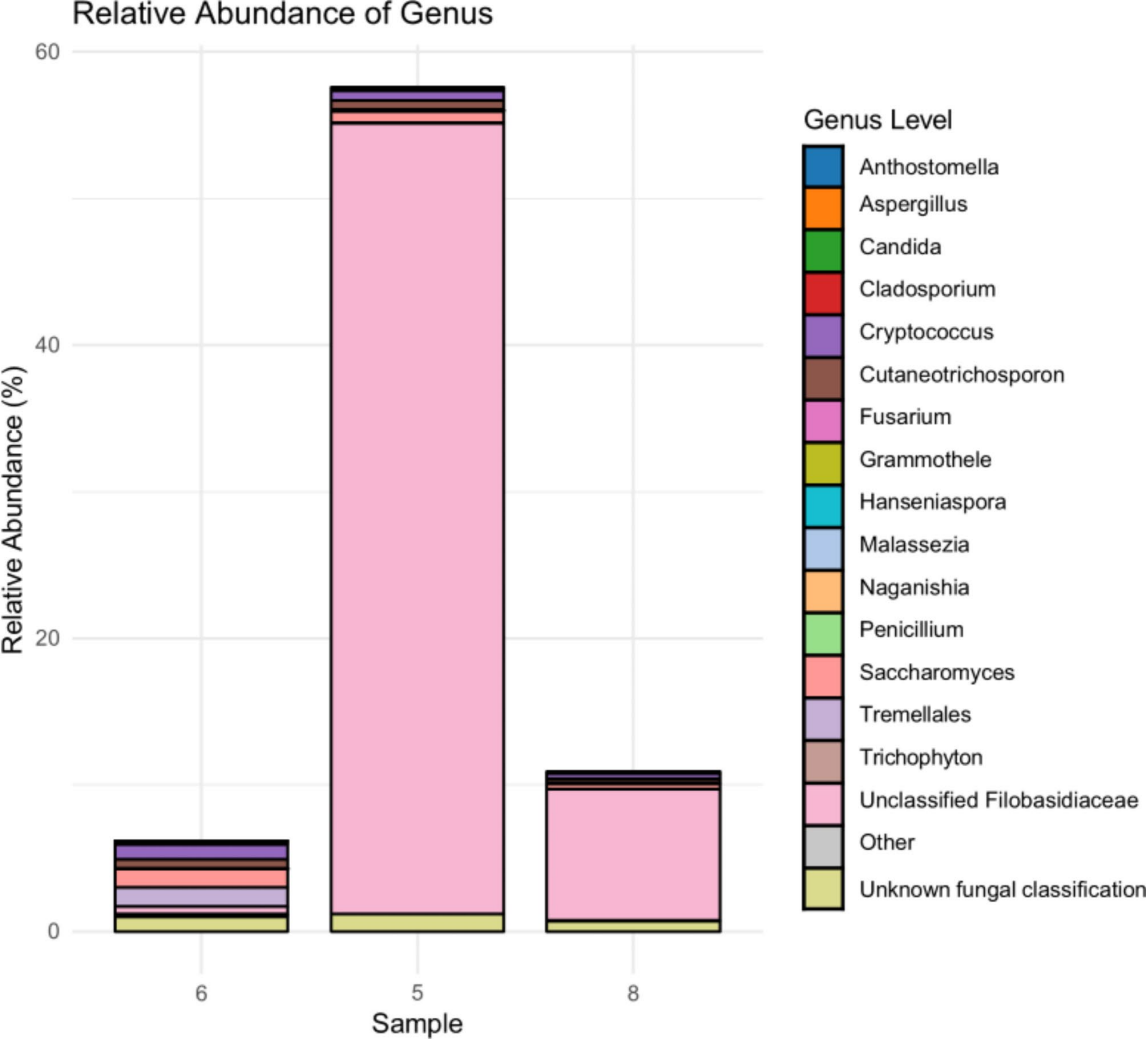
Fungal relative abundance at genus level at three samples (Barcodes 5—AD brain, 10,171 reads; 6—control brain, 7543 reads and 8—AD brain, 12,307 reads) that had the highest burden of fungal DNA. Colours indicated classification of reads across the top 20 most abundant genera.

The only human brain samples that contained a higher number of reads were barcodes 5 (AD brain, 10171 reads), 6 (control brain, 7543 reads) and 8 (AD brain, 12307 reads) (Fig. 2A represented by the black circular dot and Supplementary Table S1). Figure 3 depicts the relative abundances for barcodes 5, 6 and 8 with the exclusion of the human reads, allowing for a closer focus on the fungal burden in those samples. The total number of reads for barcodes 5, 6 and 8 was 10,149, 7543 and 12,307, respectively (Supplementary Table S1); however, when removing human DNA from the analysis we obtained a relative abundance (fungi only) of 57.8%, 6.2%, and 10% for barcodes 5, 6 and 8 respectively. Barcode 5 relative abundance is higher than barcode 6 and 8, as observed in Fig. 3. When looking at barcode 5 it is worth pointing out that 54% (relative abundance) of the reads were classified as “Unclassified Filobasidiaceae” whereas for barcode 8 it is only 8.9% (Supplementary Table S1). A common trend of barcodes 5, 6 and 8 is the presence of members of the genera *Cryptococcus*, *Saccharomyces* and *Cutaneotrichosporon*. In addition, we detected the presence of *Candida* in barcode 6 and 8 but not in barcode 5.

## Discussion

AD and PD are both neurodegenerative diseases associated with the abnormal aggregation of proteins in the CNS such as amyloid-β, tau and α-synuclein^48^. Despite the fact that both AD/PD have been studied for over a century, the exact causes of these neurodegenerative diseases are still far from being understood^49^. Several studies have been able to establish a link between bacterial or viral infection and AD/PD^50,51^, however, few have attempted to establish a connection between fungal infection and AD/PD. Studies conducted by Pisa and Alonso et al.^17–22^ have detected fungal DNA in AD/PD brains belonging to species in the *Alternaria*, *Botrytis*, *Candida*, *Cladosporium*, *Malassezia*, *Saccharomyces*, and *Cryptococcus* genera, with the latter being the most commonly found genus. *Alternaria*, *Candida*, *Cladosporium*, *Malassezia*, and *Cryptococcus* species have been found to be capable of infecting the CNS and are capable of causing fungal menigitis^16,42^. In fact, there have been reports of cryptococcal meningitis misdiagnosed as AD due to its cause of dementia-like symptoms in patients, who demonstrated loss of memory and independence in performing certain activities as well as slight behavioural changes, with these cognitive deficits reversed following antifungal treatment^52,53^.

Here, we used standard PCR-based methods alongside nanopore sequencing on the MinION platform to profile the microbial content of healthy and AD/PD brains and CSF. First, we developed and validated a method allowing the detection of fungal DNA in mammalian brain tissue. Primers targeting the fungal ribosomal operon with an amplicon size of 3.5 kb were used, followed by nanopore sequencing, as well as qPCR and RT-qPCR (Fig. 1; Table 2). qPCR and RT-qPCR did not detect fungal DNA in any human brain/CSF samples. The qPCR assay employed in this study allowed the detection low levels of fungal DNA in mice brain (Table 3). However, the number of spores spiked into the mice brains is not proportional to genome copies in the mycobiome standards and there is a ~ 1-fold loss of sensitivity. Possible explanations for this include (i) when counting the number of spores under the microscope there is always some error in accuracy, (ii) some spores may have lost viability and their DNA may have degraded, (iii) the fungal cell wall is resilient to our DNA extraction process and we may have extracted fewer fungal spores than initially counted. Future work would focus on exploring ways to enhance the efficiency of extracting DNA from fungal-infected host tissue.

Secondly, we used MinION sequencing to detect and quantify fungal species present in brain tissue samples. Our nanopore approach successfully identified all ten genera (barcode 11; Fig. 2A) present in the mycobiome genomic standards: *Alternaria*, *Aspergillus*, *Candida*, *Cryptococcus*, *Cutaneotrichosporon*, *Fusarium*, *Malassezia*, *Nakaseomyces* (*Nakaseomyces glabratus* previously known as *Candida glabrata*), *Penicillium*, *Saccharomyces* and *Trichophyton*. In addition, our nanopore approach was able to detect the three species (barcode 19) spiked into the mice brain, although for both barcodes (11 and 19) the number of reads for the genus *Aspergillus* is extremely small. This might be explained due to the large sequence divergences within this region^54^ and also due to errors in sequences annotations over the years that could lead to false assumptions when it comes to species identification.

Of relevance, our findings suggest the presence of fungal DNA in two AD patients (barcode 5 and 8) and one control brain (barcode 6) with the genera *Cryptococcus*, *Saccharomyces* and *Cutaneotrichosporon* being the 3 most common genera found amongst the three patient samples. This finding is in line with studies conducted by Pisa and Alonso et al.^17–22^. In addition, it is worth noting that barcode 6 was used on a control brain sample from a 90-year-old patient that exhibited an extensive amyloid angiopathy, which is characterized by the accumulation of amyloid β-peptide. The presence of fungal DNA in barcode 6 could be expected as AD disease is associated with the abnormal aggregation of proteins such as amyloid β-peptides. Furthermore, it can be observed that barcodes 5, 6 and 8 have reads classified as members belonging to the family Filobasidiaceae. This family previously contained (before the move to “one fungus, one name”) fungi that are known human pathogens such as *Cryptococcus neoformans. C. neoformans* is known to be able to capable of crossing the BBB and to cause meningitis^55,56^. However, we cannot assume that the fungal reads classed as “Unclassified Filobasidiaceae” are *Cryptococcus* or other species or genera that used to belong that family, since there were changes in taxonomy and there are still possible lapses in the fungal database that were not updated. The lower sensitivity found in our nanopore approach when compared to the qPCR assay (FungiQuant), is further supported by a study where they aimed to detect bacterial biodefense pathogens by using amplicon sequencing. In this study it was found that after 10 min of nanopore sequencing the LODs were at least one to two orders of magnitude lower than qPCR (single plex)^57^.

In the future to improve sensitivity and confidence of species identification in metabarcoding studies, multiple pipelines and databases may need to be used. In addition, newer machine learning-based classification systems may also be considered to enable the identification of novel species^58^. Use of fungi-specific primers for PCR also reduces the need for depletion of background or host DNA and can increase detection of species with lower abundances^59^. This is however accompanied by the drawback of PCR artefacts and biases which affect the estimation of species abundance as the use of universal primers produces different amplification efficiencies in different species^59^. Varying copy numbers of the ribosomal operon across species may also affect the number of reads obtained from each species and skew abundance estimations^59^.

In conclusion, we found evidence of the presence of fungal DNA in one healthy human brain (with extensive amyloid angiopathy), as well as in two AD brains by the use of long-read nanopore sequencing. The approach developed during this study to detect low levels of fungal DNA in CNS tissue will be of use for further exploring the microbial aetiology hypothesis for AD and PD.

## Electronic supplementary material

Below is the link to the electronic supplementary material.

Supplementary Material 1

Supplementary Material 2

## Data Availability

All raw reads have been submitted to the National Center for Biotechnology Information with accession number: PRJNA1048355.Code availabilityAll scripts can be found at https://github.com/harrychown/brains/.

## Code availability

All scripts can be found at https://github.com/harrychown/brains/.
