# Supplementary material for: Detection of fungal sequences in human brain: rDNA locus amplification and deep sequencing
Source: Sci Rep. 2024 Dec 30;14:31790. doi: 10.1038/s41598-024-82840-7 (PMC11685392; doi:10.1038/s41598-024-82840-7)
Supplement: Supplementary file 1 — Supplementary Material 1 [file 41598_2024_82840_MOESM1_ESM.docx]

Supplementary Information (Leitao *et al*., 2024)

**Supplementary Information**

**Detection of fungal sequences in human brain: rDNA locus amplification and deep sequencing**

**Rodrigo Leitao, Iam Ut Wan, Harry Chown, Thomas J Williams, Matthew C. Fisher and Johanna Rhodes**

**Supplementary File S1. DNA extraction method and validation qPCR Limit Of Detection (LOD)**

Five different DNA extraction methods were tested during this study, in order to understand which method would be the most efficient at extracting genomic DNA from mammalian brain tissue. A mock community of three fungal species (*Aspergillus fumigatus*, *Candida albicans* and *Cryptococcus* *neoformans*) was used as positive control and total of 600 spores were spiked into mice brains. Mice brains were incubated at 37°C for 17 hours in a rotatory incubator at 300 rpm. Brains were centrifuged at maximum speed and the supernatant was discarded. DNA extractions were performed following the five protocols:

1. DNeasy Blood & Tissue Kit with no bead beating

2. DNeasy Blood & Tissue Kit with beading (PowerBead Pro Tubes, Cat. No 19301 before addition of Proteinase K (Step 2)

3. DNeasy Blood & Tissue Kit with beading (PowerBead Pro Tubes, Cat. No 19301) after addition of Proteinase K (Step 3)

4. DNeasy PowerSoil Pro Kits (Qiagen, Germany, 47014)

5. ZymoBIOMICS DNA Miniprep Kit (D4300)

Average Cq values from FungiQuant (qPCR assay) were used to determine which method performed better in relation to the others. The DNA extraction method that amplified earlier on the qPCR was selected. DNeasy PowerSoil Pro Kits performed better than ZymoBIOMICS DNA Miniprep Kit (method 5) and DNeasy Blood & Tissue Kit (method 1) with Cq values of: 28.84; 29.14 and 34.59 respectively. Method 2 and 3 did not perform as well as the others and were removed from the analysis. Due to the earlier amplification on the qPCR assay (Cq values) we have decided to use the DNeasy PowerSoil Pro Kit for our study.
